# Famous People Knowledge and the Right and Left Temporal Lobes

**DOI:** 10.3233/BEN-2012-0347

**Published:** 2011-12-29

**Authors:** Julie S. Snowden, Jennifer C. Thompson, David Neary

**Affiliations:** ^1^Cerebral Function UnitGreater Manchester Neuroscience CentreSalford Royal Foundation TrustSalfordUK; ^2^Mental Health and Neurodegeneration Research GroupFaculty of Medicine and Human SciencesUniversity of ManchesterManchesterUK

**Keywords:** Semantic dementia, anterior temporal lobes, semantic hub, amodal, modality, person knowledge

## Abstract

It is generally accepted that the anterior temporal lobes support knowledge of famous people. The specific roles of the right and left temporal lobe remain a subject of debate, with some studies suggesting differential roles based on modality (visual versus verbal information) and others category (person knowledge versus general semantics). The present study re-examined performance of semantic dementia patients with predominantly right and predominantly left temporal lobe atrophy on famous face, famous name and general semantic tasks, with the specific aim of testing the hypothesis that the right temporal lobe has a privileged role for person knowledge and the left temporal lobe for general semantic knowledge. Comparisons of performance rankings across tasks showed no evidence to support this hypothesis. By contrast, there was robust evidence from naming, identification and familiarity measures for modality effects: right-sided atrophy being associated with relatively greater impairment for faces and visual tasks and left-sided atrophy for names and verbal tasks. A double dissociation in test scores in two patients reinforced these findings. The data present a challenge for the influential ‘semantic hub’ model, which views the anterior temporal lobes as an area of convergence in which semantic information is represented in amodal form.

## 1. Introduction

There is good evidence that the anterior temporal lobes support knowledge about famous people. Impairments in recognising and naming famous people have been demonstrated in patients with temporal lobe lesions and following temporal lobe resection [4,10,45, 46]. Famous faces have been shown to elicit activation in the anterior temporal lobes in functional imaging studies [3,11,35,42,47].

A compelling source of evidence comes from patients with semantic dementia (SD) [14,15,38–40], a degenerative disorder of the anterior temporal lobes that is associated with severe, multimodal loss of conceptual knowledge of the world. The disorder affects the ability to name and comprehend words, to recognise the significance of objects, non-verbal sounds, tastes, smell and tactile stimuli. Importantly, a ubiquitous and striking early feature is loss of knowledge of famous people.

In 2004 we carried out a comparative study of famous person knowledge in SD patients with anterior temporal lobe atrophy and amnesic Alzheimer (AD) patients with medial temporal lobe atrophy [41]. Both patient groups were impaired compared to controls in naming famous faces and in providing identifying information about famous people from their face and from their name. However, SD patients were considerably more impaired than the AD patients. Moreover, only the SD patients were also significantly impaired on forced-choice familiarity judgement tasks requiring selection of the famous face or name from a famous/non-famous pair. Consistent with this finding, subjective reports of familiarity were significantly lower compared to con-

ISSN 0953-4180/12/$27.50 © 2012 – IOS Press and the authors. All rights reserved trols in the SD patients but not in the AD patients. Such differences between SD and amnestic AD could not be attributed to overall differences in severity of illness. The two groups were matched for age, duration of illness and Mini-Mental State scores. Moreover, on conventional measures of episodic memory (orientation in time and place and object recognition memory) it was the AD patients who were significantly more impaired. The findings provide strong evidence of the importance of the anterior temporal lobes in supporting knowledge of famous people.

An unresolved issue is the precise role of the two hemispheres in person knowledge. Some studies have identified modality specific deficits for famous faces and names as a function of laterality of lesion [5–8,18] and modality specific activations for faces and names on functional neuroimaging [47] suggesting differential contributions of the two hemispheres to person-knowledge based on modality. However, other studies have suggested a role for the right temporal lobe in cross-modal person knowledge [2,4,13,19].

SD provides a unique opportunity to address putative functional differences between left and right temporal lobe, because the temporal lobe atrophy, although bilateral, is often highly asymmetrical. In our own study [41] patients with predominantly left-sided atrophy ($n = 10$) were better at providing identifying information about famous faces than their corresponding names, whereas patients with predominantly right-sided atrophy ($n = 3$) showed the opposite pattern. A similar left/right dissociation was demonstrated for forced-choice and subjective familiarity judgements. Thus, stimulus modality was a significant determinant of performance. We interpreted the findings as evidence for the differential importance of the left and right temporal lobes respectively for verbal and visual semantic information.

Findings from a contemporaneous study by the Cambridge group of two SD patients [43] were complementary in so far as they illustrated the importance of the anterior temporal lobes in famous person knowledge and demonstrated differential roles of the left and right temporal lobes. However, there were fundamental differences. In that study one patient, MA, with predominantly left sided atrophy was more impaired than the second patient, JP with predominantly right-sided atrophy on the Pyramids and Palm trees test of semantic association, both in its word and pictorial form. By contrast, JP was more impaired on methodologically similar associative matching tasks involving famous faces and names. That is, performance in the two patients dissociated on categorical grounds and not stimulus modality. The findings led the authors to argue that the left temporal role is particularly important for 'general semantics' and the right temporal role for person knowledge.

The different conclusions of the two studies have considerable theoretical importance. Contemporary models view semantic memory in terms of a widely distributed cerebral network [1,26,29,44]. It is acknowledged that conceptual knowledge of an object includes knowledge of its visual and other sensory attributes, its mode of action and its name. The semantic network is thought to be organised, at least in part, in accordance with corresponding sensory, motor and language systems. A more contentious issue is whether there exists, in addition to this modal network, a 'semantic hub' located in the anterior temporal lobes, which acts as an area of convergence giving rise to conceptual representations that are independent of modality, and represented in amodal form [29,33,34]. Such a hub is necessary, it is argued [23,29] to achieve the higher-order generalisations across concepts that are a fundamental and defining feature of semantic memory. It permits objects that are very different in terms of their perceptual characteristics, mode of manipulation and name to be recognised as semantically-related. The profound semantic impairment found in SD patients that cuts across sensory modalities has been interpreted as evidence of an amodal disorder reflecting damage to this anterior temporal lobe semantic hub [23,29]. Performance differences in SD patients as a function of category (object semantics vs. person knowledge), as found by Thompson et al. [43] would potentially be compatible with an 'amodal representation' account, whereas performance differences as a function of stimulus modality (visual vs. verbal) would not.

In view of its theoretical implications the issue of the differential role of the left and right temporal lobe requires further exploration. The present study re-examined the data reported by us in 2004 [41] with the specific aim of testing the hypothesis, proposed by Thompson et al. [43], that the right temporal lobe has special importance for person-knowledge *per se*. That is, it sought to determine whether there was evidence for categorical as opposed to modality-specific explanations of left-right differences in SD. Accordingly patients' performance on famous face/name tasks was compared to that on general semantic tasks. A general limiting factor in studies of SD is that patients with predominantly right-sided atrophy are exceedingly rare. Since the 2004 study we have had the opportunity to

Table 1
Background neuropsychological data on individual SD patients

| Atrophy | Right > Left | | | | Left > Right | | | | | | | | | |
|---|---|---|---|---|---|---|---|---|---|---|---|---|---|---|
| Patients* | 1 | 2 | 3 | 4 | 1 | 2 | 3 | 4 | 5 | 6 | 7 | 8 | 9 | 10 |
| MMSE /30 | 27 | 27 | 18 | – | 27 | 26 | 23 | 25 | 24 | 21 | 25 | 20 | 6 | 7 |
| Graded naming /30 | 0 | 0 | 1 | 0 | 0 | 3 | 1 | 0 | 0 | 0 | 0 | 0 | 0 | 0 |
| Manchester naming /40 | 27 | 31 | 29 | 0 | 32 | 34 | 33 | 28 | 12 | 17 | 14 | 0 | 9 | 0 |
| Word-picture match /40 | 33 | 40 | 40 | 5 | 40 | 40 | 40 | 40 | 31 | 37 | 38 | 10 | 36 | 8 |
| P & P pictures /52 | 30 | 41 | 39 | 25 | 48 | 48 | – | 45 | 32 | 39 | 39 | – | – | – |
| P & P words /52 | 46 | 43 | 45 | 26 | 46 | 43 | – | 38 | 26 | 32 | 29 | – | – | – |
| VOSP screen /20 | 20 | 20 | 19 | 19 | 19 | 19 | 20 | 20 | 20 | 20 | 20 | 19 | 20 | 20 |
| VOSP incomplete letters /20 | 16 | 20 | 18 | 4 | 20 | 20 | 20 | 19 | 19 | 20 | 19 | 0 | 19 | 17 |
| VOSP silhouettes /30 | 2 | 8 | 8 | 2 | 10 | 14 | 15 | 13 | 3 | 4 | 10 | 0 | 14 | 0 |
| VOSP object decision /20 | 16 | 18 | 11 | 12 | 16 | 20 | 17 | 16 | 13 | 19 | 12 | 13 | 19 | 10 |
| VOSP dot counting /10 | 10 | 10 | 10 | 10 | 10 | 10 | 10 | 10 | 10 | 10 | 10 | 10 | 10 | 10 |
| VOSP position discrim./20 | 20 | 20 | 19 | 20 | 20 | 20 | 20 | 20 | 20 | 19 | 19 | 20 | 20 | 20 |
| VOSP number location /10 | 10 | 10 | 9 | 10 | 10 | 10 | 10 | 10 | 10 | 10 | 10 | 10 | – | 10 |
| VOSP cube analysis /10 | 9 | 10 | 9 | 9 | 10 | 9 | 9 | 9 | 10 | 9 | 9 | 9 | – | 10 |
| Picture recall /20 | 0 | 1 | 3 | 7 | 7 | 1 | 0 | 7 | 1 | 2 | 3 | 0 | 0 | 0 |
| Picture recognition/20 | 10 | 18 | 10 | 16 | 18 | 20 | 18 | 14 | 12 | 16 | 16 | 15 | 19 | – |
| Orientation/10 | 10 | 9 | 6 | – | 10 | 9 | 8 | 10 | 9 | 10 | 9 | 8 | – | – |

*Patient numbers do not correspond with those in the figures, which refer to performance rankings.

study a new SD patient with predominantly right-sided atrophy permitting data from that additional patient to be included in the present study.

The face and name tasks used in the current study are matched and therefore scores can be directly compared and item-by-item correspondences can be calculated. Person tasks are not, however, matched for difficulty with general semantic tasks, so that comparison of absolute test scores is of limited value. Nevertheless, the primary feature of interest in the study is not absolute performance, but rather *relative* performance in right and left predominant patients for person-semantic and general semantic tasks. If the right temporal lobe is specialised for person based knowledge and the left temporal lobe for general semantics then this should be manifest by *relative* differences in performance, as determined by rankings of scores, in patients with more right and more left sided atrophy on person semantic and general semantic tasks.

## 2. Participants

The participants comprised 14 patients with a clinical diagnosis of SD, 8 men and 6 women, who attended a specialist early-onset dementia clinic. The criteria for selection of patients was that a) they had undertaken an assessment of famous face and name knowledge, b) background data on general semantic tasks were available and c) they showed an asymmetrical distribution of temporal lobe atrophy on MR imaging. Thirteen of the patients had participated in the face/name study reported previously [41], whereas one had not. Atrophy was more marked on the right side in four patients, and on the left side in 10 patients. The mean age at the time of assessment in the right and left-sided groups was respectively 67.5 (s.d. 6.2) and 65.5 (s.d. 6.0) years, which did not differ significantly. The mean duration of illness was 4.8 (s.d. 3.4) and 4.1 (s.d. 1.4) years, which did not differ significantly. Patients' performance on background semantic and other neuropsychological tests is shown in Table 1.

## 3. Materials and methods

### *3.1. Famous faces and names*

The famous person assessment involved 75 photographs of famous people and their corresponding names as described previously [41]. Outcome measures comprised:

i) Naming: uncued naming of famous faces
ii) Identification: provision of descriptive, identifying information by the patient of a) faces and in a separate, subsequent task b) their corresponding names
iii) Familiarity: forced-choice judgement based on selection of a) the famous face and b) in a separate subsequent task, the famous name from a famous/non-famous pair.
iv) Subjective familiarity: report from the patient whether responses in the forced-choice judgement face and name tasks were guesses or based on feelings of familiarity.

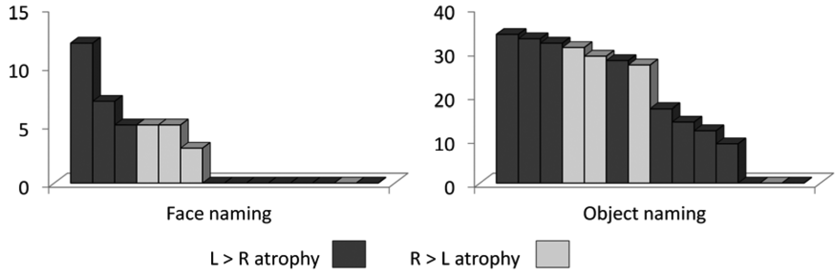

Fig. 1. Naming performance in individual SD patients on famous face and object naming tasks, ranked in order of test scores. Patients with predominant right and left temporal lobe atrophy are distinguished by shading.

### 3.2. General semantic tasks

Data from general semantic tasks were available from patients' background neuropsychological assessment. These tasks had not initially been designed for the purpose of comparison of with face/name tasks so are not precisely equivalent methodologically. Nevertheless, they tap the same broad levels of knowledge: naming (picture naming), identity (recognising the significance of pictures and words) and familiarity (judging which one of four shapes is a real object). Patients' data from the following general semantic tasks were used for comparative purposes:

i) Naming: a locally developed picture naming test [41].
ii) Identification: Pyramids and Palm trees test of semantic association [16], a) pictorial and b) word version; c) Silhouettes subtest of Visual Object and Space Perception Battery (VOSP) [49].
iii) Familiarity: Object Decision subtest of VOSP

Data from the Graded Naming test [27] were not used in the analyses because floor level scores precluded meaningful ranking of performance.

### 3.3. Procedure

The aspect of data of most interest was the *relative* performance of patients with more right-sided atrophy compared to those with more left-sided atrophy on famous person versus general semantic tasks. Accordingly, patients were ranked in order of their test scores.

### 3.4. Statistics

In order that equivalent comparisons could be made for cross-category (person semantics vs. object semantics) and cross-modality (visual vs. verbal) performance statistical comparisons were based on individual performance ranks. Mann Whitney U tests, comparing SD patients with right and left temporal atrophy), were conducted on differences in patients' ranks for two tasks (e.g. famous face naming ranks – picture naming ranks). Two-tailed test results are reported.

Item-by-item correspondence in cross-modality comparisons for individual patients was assessed by McNemar's test for the significance of changes.

## 4. Results

### 4.1. Comparison of performance in patients with right compared to left-sided atrophy based on rankings of test scores (*relative performance rather than absolute test scores*)

#### 4.1.1. Naming: Faces, objects

Performance of individual patients on face and object naming tasks is shown in Fig. 1, ordered in terms of naming scores. On face naming, three patients with right-sided atrophy performed in the middle range for the cohort, their scores being ranked 4,5 and 6. The remaining right-sided patient scored at floor level, along with seven left-sided patients. The object naming test elicited a wide range of scores across the cohort, ranging from 85–0% correct. Scores for the three right-sided patients again fell within the middle of the range, being ranked 4,5 and 7, whereas the remaining right-sided patient performed at floor level. Differences in ranking for faces and object (face naming rank – object naming rank) did not differ in the two SD sub-groups (patients with greater right-sided atrophy vs. patients with greater left-sided atrophy) (U = 15.5, $z = -0.648$, $p = 0.52$). Therefore, naming tasks provided no evidence for *relative* performance differences for face versus object naming in patients with right compared to left-sided atrophy.

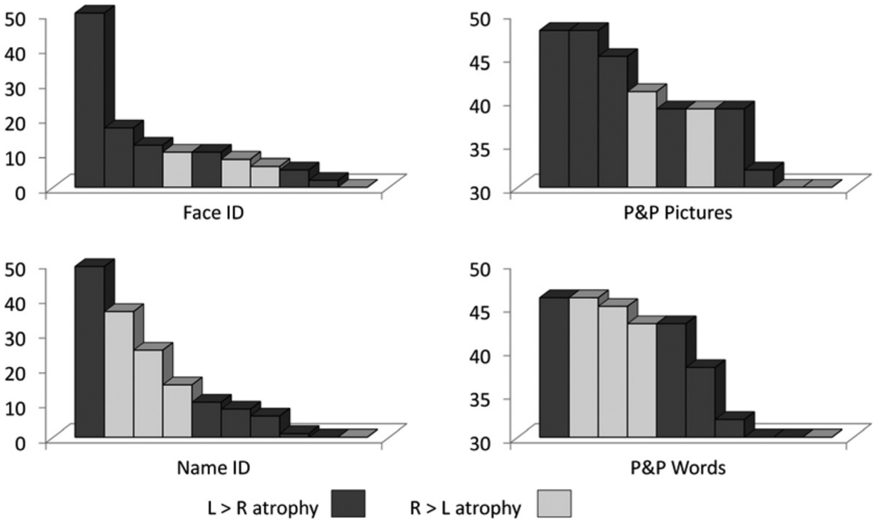

(a)

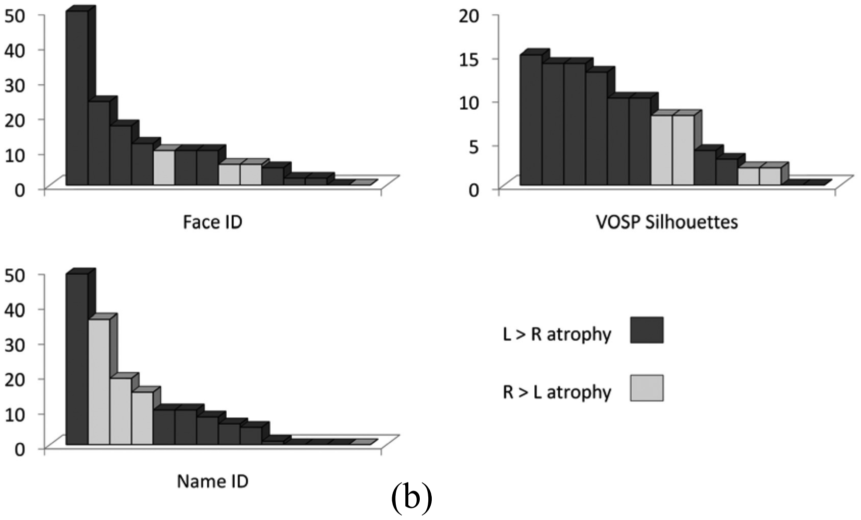

(b)

Fig. 2. (a) and (b) Identification performance in individual SD patients on famous face, famous names and object-knowledge tasks, ranked in order of test scores. Patients with predominant right and left temporal lobe atrophy are distinguished by shading.

*4.1.2. Identification: Faces, names, objects, words*

Identification performance for individual patients on famous face and name tasks is shown in Fig. 2a, ranked in order of test scores. Performance is also shown for object and word association scores on the Pyramids and Palm trees test of semantic association. Data were available for only 10 patients on the Pyramids and Palm trees test, so person-object knowledge comparisons are based on these 10 patients only. The figure shows that face identification scores for 3 patients with right-sided atrophy fell within the middle range for the cohort, being ranked 4,6 and 7, whereas the remaining right-sided patient performed at floor level and ranked 10. Rankings of performance in these patients on the pictorial version of the Pyramids and Palm trees test were similar (4,6,9 and 10). Differences in rankings between face and picture tasks were not significant (U = 10.5, $z = -0.33$, $p = 0.75$). However, rankings improved for verbal tasks in all but the most severely affected patient who performed at floor level. Rankings were 2,3, 4 and 10 on both famous name identification and the word version of the Pyramids and Palm trees test. Differences in rankings for face and name identification strongly distinguished the two SD sub-groups (U $= 0.00$, $z = -2.57$, $p = 0.01$). Differences in rankings for picture and word versions of the Pyramids and Palm

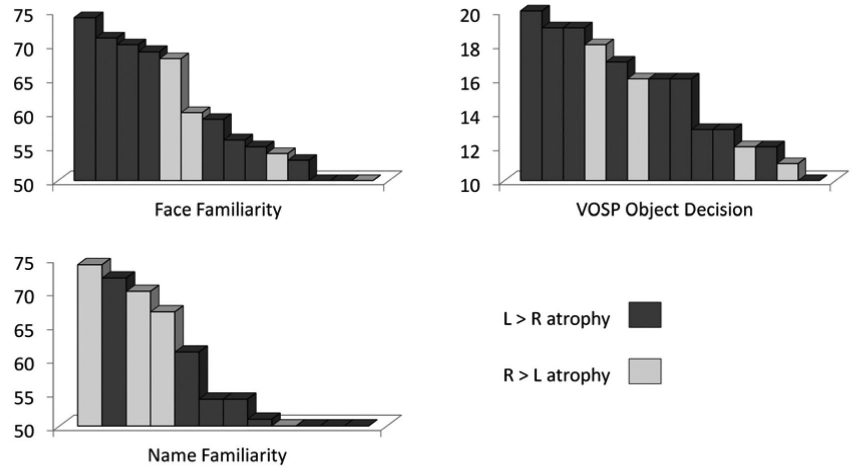

Fig. 3. Familiarity performance in individual SD patients for famous faces, famous names and objects, ranked in order of test scores. Patients with predominant right and left temporal lobe atrophy are distinguished by shading.

trees test approached significance (U $= 3.5$, $z = -1.86$, $p = 0.06$). However, differences in rankings for famous name identification and the Pyramids and Palm Trees word task were not significant (U $= 7.00$, $z = -1.12$, $p = 0.26$).

Identification performance on famous face and name tasks is shown for all 14 patients in Fig. 2b, together with performance on the VOSP silhouettes test (a task requiring object identity). Performance in right-sided patients was ranked 5,8,9 and 14 on the Face identity task, and 7,8,11 and 12 on the Silhouettes test. Differences in rankings for face and object tasks were not significant (U $= 18.5$, $z = -0.22$, $p = 0.84$). By contrast, performance in right-sided patients improved on the famous name identification task, rankings being 2,3,4 and 14. Differences in rankings for famous face and name tasks were highly significant (U $= 0.5$, $z = -2.77$, $p = 0.006$).

In summary, examination of *relative* performance in patients with right compared to left-sided atrophy indicates no evidence of a notable shift in rankings as a function of semantic category. Specifically, there is no evidence of proportionally greater difficulty in right-sided patients for person semantics as opposed to object semantics. By contrast, there is a notable shift in rankings as a function of modality. Patients with right-sided atrophy show improved performance, relative to other patients in the cohort, on verbal compared to visual semantic tasks, irrespective of semantic category.

*4.1.3. Familiarity: Faces, names, objects*

Individual performance on the forced-choice familiarity judgement task for famous faces and names is shown in Fig. 3, ranked in order of test scores. Performance is shown for comparison on the VOSP object decision task, which requires recognition of a shape as a real object and therefore depends on object familiarity. Three right-sided patients performed in the middle range for the group on the forced-choice famous face task, whereas the fourth performed at floor level. Scored were ranked 5,6,10 and 14. There was no improvement in rankings for object decision test performance, scores being ranked 4, 6, 11 and 13. Differences in rankings for face and object familiarity tasks were not significant (U $= 19.5$, $z = -0.07$, $p = 0.94$). By contrast, there was an improvement in rankings on the forced-choice task involving famous names, scores for right-sided patients being ranked 1,3,4 and 9. Differences in rankings for famous face compared to famous name familiarity was statistically significant (U $= 2.0$, $z = -2.57$, $p = 0.01$).

The shift in rankings for the famous name compared to the famous face task is mirrored by changes in explicit reports of subjective familiarity (whether patients felt responses to be based on actual feelings of familiarity or guesses). The most severely affected patient denied feelings of familiarity for all items, both faces and names so no differential performance was elicited. However, the remaining three patients showed a relative increase in subjective familiarity reports for names compared to faces, the percentage of familiarity reports being ranked 7, 9 and 10 for faces, but 1, 3 and 4 for names. The difference in rankings for face and name subjective familiarity reports was statistically significant (U $= 0.00$, $z = -2.89$, $p = 0.004$).

In summary, examination of performance ranks for patients with more right-sided atrophy showed no con-

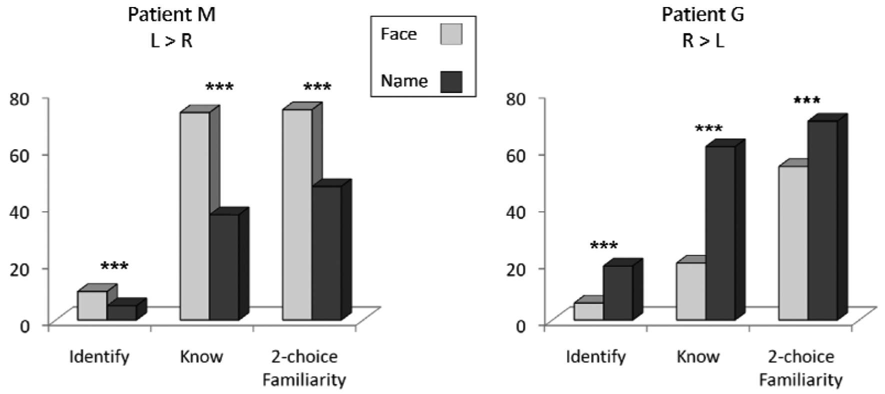

Fig. 4. Scores on famous face and name identification, two-choice familiarity judgement, and subjective familiarity (know) in patient M with predominant left-sided temporal lobe atrophy and patient G with predominant right-sided atrophy. *** p $\leqslant$ 0.001 item-by-item comparison for faces and names, McNemar test.

vincing evidence of differential relative performance in familiarity judgements for person compared to object-based tasks. By contrast, their performance, improved for name compared to face tasks relative to patients with left-sided atrophy.

### 4.2. *Evidence of double dissociations in performance in patients with left versus right-sided atrophy*

Examination of individual test scores showed no instances of double dissociation as a function of category, as reported by Thompson et al. [43]. By contrast, there were instances of dissociated performance as a function of modality. Figure 4 shows actual scores on famous face and name identification, two-choice familiarity judgement, and subjective familiarity in patient M with more left-sided temporal lobe atrophy and patient G, with more right-sided atrophy. The patients are both female, are similar in age (61 vs. 60 years) and have a similar duration of illness (3 years). Item-by-item comparisons using McNemar tests shows, in patient M, significantly better identification ($p < 0.001$), forced-choice judgement ($p < 0.001$) and subjective familiarity ($p < 0.001$) for famous faces than the corresponding famous names. Patient G shows the converse pattern. Performance is significantly better for names than faces, for identification ($p = 0.001$), forced-choice judgement ($p < 0.001$) and subjective familiarity ($p < 0.001$).

## 5. Discussion

The study was motivated by current debate regarding the role of the anterior temporal lobes and in particular by putative differential roles of the left and right temporal lobe. The study examined specifically whether there is evidence from SD patients for a privileged role of the right temporal lobe in the representation of person-knowledge as opposed to general semantic knowledge, as suggested by Thompson et al. [43]. The hypothesis is plausible in view of a wealth of data suggesting that person knowledge is dissociable from other categories of semantic knowledge [5,9,17,20,24,25,28,37], and therefore may be subserved by distinct neural systems. It is worth noting, however, that loss of person-knowledge has not invariably been ascribed to right, as opposed to left-temporal damage [9].

The study failed to find evidence in the SD cohort of greater relative impairment of person-knowledge in patients with predominant right-sided temporal atrophy and object-semantics in patients with predominant left-sided atrophy. There were no instances of dissociations in performance on famous person and other semantic tasks of the kind reported by Thompson et al. [43]. By contrast, there was strong evidence of modality effects, with poorer relative performance in patients with right-sided atrophy for visually based tasks, irrespective of task type, and poorer relative performance for verbal tasks in patients with left-sided atrophy. Moreover, a compelling double dissociation in performance was elicited for famous faces and their corresponding names in two patients, G and M, with predominant right and left-sided atrophy respectively.

One prevailing explanation of modality effects in SD is that visual/verbal tasks are not of comparable difficulty. SD patients frequently recognise objects while failing to recognise their names. A cogent argument to account for this observation [22] is that the visual properties of objects provide clues to their function, whereas object labels do not. In a degraded semantic system the available information may be sufficient to support object but not word recognition. Such an argument, while entirely reasonable, has difficulty accounting for differential performance for famous faces and names since both are arbitrary and provide little inherent information about the person's identity. Moreover, it would not predict the relative superiority of performance for names compared to faces in patients with right-sided atrophy, nor double dissociations in face/name performance for patients with right and left-sided atrophy. Our data provide compelling evidence for modality effects in semantic dementia, which are not an artefact of task difficulty.

The notion that the left and right temporal anterior lobes both contribute to famous person knowledge, albeit differentially, accounts for the fact that unilateral, focal lesions rarely produce the profound and pervasive loss of semantic knowledge that characterises SD. The point is well illustrated by a study of Lambert et al. [21], which explored person-related knowledge in a mixed cohort of patients with left and right temporal lesions, including 2 with left and 3 right temporal lobectomy. Greater face recognition impairment was elicited in patients with right sided lesions and greater naming impairment in patients with left sided lesions, but no general person-specific knowledge impairment was demonstrated in either group. The authors argued that bilateral lesions are necessary for such generalised knowledge loss. The requirement for bilateral contribution would also account for the evolution of semantic loss in a patient with right-predominant degeneration described by Evans et al. [5]. A relatively circumscribed face recognition disorder progressed to a cross-modal semantic impairment and involvement of other semantic categories. In SD the degenerative process is invariably bilateral, albeit with marked asymmetries.

The finding of modality effects in SD is in keeping with findings of Gainotti and colleagues. In one study [7] these authors reported a patient with right temporal atrophy who showed impaired access to semantic knowledge about famous people from their face and voice but not from verbal description. In another study [8] they demonstrated dissociations in familiarity judgements for faces and names in two patients with right and left temporal atrophy.

The influential 'semantic hub' view of semantic memory proposes that the anterior temporal lobes represent an area of convergence where concepts are represented in amodal form [29,34]. Recent evidence that transcranial magnetic stimulation, applied either to the left or right temporal pole, disrupts semantic processing for words and pictures to a comparable degree, has been interpreted as support for the amodal hub hypothesis [31,32]. The most compelling evidence for an amodal hub, however, comes from patients with semantic dementia. Patients have profound loss of conceptual knowledge that cuts across modality: they have difficulty recognising verbal information, non-verbal environmental sounds, visual, tactile, gustatory and olfactory stimuli. Moreover, performance on semantic memory tasks assessed through different modalities is typically correlated [22]. These characteristics of SD are in keeping with the notion of amodal representation. The amodal model predicts, however, that in SD performance breakdown across modalities should *invariably* be correlated, yet this is not the case. The findings of dissociable performance as a function of modality and laterality of atrophy, found in the present study, present difficulties for the amodal account.

A putative explanation that requires consideration is whether patients with right and left-sided atrophy have additional pre-semantic perceptual or linguistic processing deficits that contribute to their overall performance. There are no compelling grounds for assuming this to be so. Patients all performed well on tests of visual perception, except when tasks required recognition of identity (semantics). They all spoke fluently and effortlessly, with no evidence of linguistic impairment outside the semantic domain. They could read out names of people that they did not recognise. Although data for elementary tests of face processing were not available in this study, SD patients in general, including those studied in this centre, show no difficulty on non-semantic face matching and discrimination tasks. All patients in the present study showed a severe, yet essentially circumscribed disorder of semantic memory.

Some authors have argued [30,48] that, even in an amodal system, activation within the anterior temporal lobes might be influenced by the modality of the task due to variations in connectivity with modality-specific areas in posterior temporal cortex. Such an argument might hold for differences, found in functional imaging studies, in the relative strength of activation in right and left anterior temporal lobe for faces and names. It has more difficulty accounting for the frank dissociations in recognition of faces and their corresponding names observed in this study in SD patients.

Alternative interpretations of the role of the anterior temporal lobes have been advanced, distinct from the semantic hub account [36]. Some authors suggest that these regions underpin knowledge of unique entities such as familiar people [3,12], hence the observed dissociations between knowledge of people and for objects and words. Other authors emphasise their role in social conceptual knowledge [50]. A recent fMRI study attempted to adjudicate between the domain-general semantic hub view and these alternatives [37]. Healthy participants learnt equivalent/matched facts (name, age, location, occupation/function) about unique but novel persons, buildings and hammers. Conjunction and region of interest analysis showed person-selective responses in both left and right anterior temporal lobe. No domain-general responses were elicited in the anterior temporal lobe to indicate its role as an amodal semantic hub. The authors argued that the anterior temporal lobes support person knowledge, and are one component in a network supporting social concepts. The relevance of the findings for the present study is the rejection of the semantic hub account. Moreover, in demonstrating the importance of both right and left temporal lobes, they counter the notion advanced by Thompson et al [43] that the right anterior temporal lobe has a special, privileged role for person knowledge. Why our data differ from those of Thompson et al. is intriguing and currently can only be speculated. However, it may be of relevance that the patient reported by Thompson et al. with relative preservation of people knowledge proved ultimately at post-mortem to have AD (personal communication from John Hodges) and not the ubiquitin-positive, TDP-43 positive histology characteristic of SD.

In conclusion, the present study found no evidence of dissociated knowledge of famous people and general semantic knowledge as a function of laterality of atrophy. The findings provided no support for the view that the right the left anterior temporal lobes have dedicated roles respectively in the representation of person knowledge and general semantics. By contrast, there was clear evidence of modality effects, face identification and familiarity being relatively more impaired in patients with predominant right temporal atrophy and name identification and familiarity more impaired in patients with predominant left temporal atrophy. Thus, even though the semantic impairment in SD is severe, pervasive and encompasses all modalities it is not modality-free. These findings are consistent with the notion that semantic knowledge is underpinned by a distributed multimodal network. They provide a challenge, however, for the view proposed by the semantic hub hypothesis, that semantic knowledge is represented in amodal form.
